# What are the initial research priorities for paediatric emergency medicine in India? A prioritisation study

**DOI:** 10.1136/bmjph-2024-001967

**Published:** 2026-06-09

**Authors:** Indumathy Santhanam, Muralidharan Jayashree, Suresh Kumar Angurana, Raghavendra Narayan Vanaki, Bharat Choudhary, Mounika V Reddy, Balaji Jayaraman, Yalaguraswami B Kolkar, Prakash Vemanna J, Siddu Charki, Akila Sivakumar, Anita Tarigopula, Jayanthi Ramesh, Arvind Yalamanchili, Pranam Gowdar, Sharada Sathish Sathishkumar, Kalpana Sivasambo, Sridevi Srinivasan, Dhakshayani Ramalingam, Priyavarthini Venkatachalapathy, Hemamalini Basker, Madhan Kumar, Madhusudan Samprathi, Aravind Anand, Ramesh Veerappa Neelannavar, Pallavi Charantimath, Franz E Babl, Stuart R Dalziel, Elliot Long, Simon Craig

**Affiliations:** 1 Madras Medical College, Chennai, Tamil Nadu, India; 2 Post Graduate Institute of Medical Education and Research, Chandigarh, India; 3 BVV Sangha’s S Nijalingappa Medical College, Bagalkot, Karnataka, India; 4 All India Institute of Medical Sciences, Jodhpur, Rajasthan, India; 5 All India Institute of Medical Sciences, Hyderabad, India; 6 Government Dharmapuri Medical College, Dharmapuri, Tamil Nadu, India; 7 BLDEA’s Shri BM Patil Medical College Hospital and Research Centre, Bijapur, Karnataka, India; 8 Rainbow Children’s Healthcare Society, Hyderabad, Telangana, India; 9 Apollo Children’s Hospital, Chennai, Tamil Nadu, India; 10 International Center for Child and Public Health, Coimbatore, Tamil Nadu, India; 11 Navodaya Medical College Hospital and Research Centre, Navodaya Nagar, Karnataka, India; 12 Mehta Multispeciality Hospitals India Pvt Ltd, Chennai, Tamil Nadu, India; 13 Government Vellore Medical College and Hospital, Vellore, Tamil Nadu, India; 14 The Tamil Nadu Dr MGR Medical University, Chennai, Tamil Nadu, India; 15 Madurai Medical College, Madurai, India; 16 Government Headquarters Hospital, Cuddalore, India; 17 Aster Whitefield Hospital, Bangalore, Karnataka, India; 18 Pediatrics, BVV Sangha’s S Nijalingappa Medical College, Bagalkot, Karnataka, India; 19 The Royal Children’s Hospital Melbourne, Melbourne, Victoria, Australia; 20 Starship Children’s Health, Auckland, New Zealand; 21 Department of Paediatrics, Monash University, Clayton, Victoria, Australia

**Keywords:** Community Health, Emergencies, Public Health

## Abstract

**Introduction:**

Paediatric emergency medicine (PEM) in India is undergoing a period of rapid growth. A key step in the establishment of successful PEM research networks in high-income countries has been prioritisation of research questions. However, these may be less relevant to India due to differences in epidemiology (higher rates of infectious disease) and limited prehospital care. This gap highlights the importance of context-specific research priorities to address disparities in resource allocation and clinical outcomes.

We report on a process to develop a prioritised list of research questions and topics aiming to improve the clinical care of children attending emergency departments across India.

**Methods:**

A nominal group technique (NGT) approach was used to set research priorities within a 1-day workshop attached to the Society for Emergency Medicine in India conference (EMCON2023) in October 2023. The workshop, which included 30 participants, was facilitated by four members of an established PEM research network from Australia and New Zealand.

The priority-setting process included idea generation and recording, open discussion and clarification of ideas and voting. The NGT method facilitated balanced participation and focused discussions, ensuring all participants’ input was equally considered. A final list of 48 prioritised research topics was generated.

**Results:**

Top-ranking areas included prehospital care, septic shock, envenomation, timing of and physiological optimisation prior to intubation, point-of-care ultrasound, the role of structured clinical assessment and trauma.

**Conclusions:**

This study has, for the first time, identified multicentre research priorities in PEM for India. The prioritisation of prehospital care reflects the urgent need to address delayed interventions, particularly in rural areas. The presented list of research questions will guide research efforts over the coming years and form the basis for the development of a multicentre research network. This initiative lays the groundwork for long-term collaboration and capacity building in PEM research across India.

**WHAT IS ALREADY KNOWN ON THIS TOPIC**

- Over the last decade, paediatric emergency medicine (PEM) networks from high-income countries have prioritised research proposals that are relevant to their settings. However, little published literature is available on research priorities for low- and middle-income countries.

**WHAT THIS STUDY ADDS**

- PEM networks in HIC settings emphasise risk stratification and diagnosis of potential life-threatening conditions, while Indian PEM physicians prioritised topics relevant to resuscitation of critically ill children. Top-ranking priorities included prehospital care, septic shock, envenomation, timing of and physiological optimisation prior to intubation, point-of-care ultrasound, the role of structured clinical assessment and trauma.

**HOW THIS STUDY MIGHT AFFECT RESEARCH, PRACTICE OR POLICY**

- This study is the first of its kind to develop a list of prioritised research questions tailored to the Indian context by PEM physicians from India. It would help obtain the much-needed evidence-based support for paediatric resuscitation protocols that have been innovated and successfully implemented within India.

## Background

 Worldwide, millions of children seek emergency care each year. Despite the large numbers of patients, there are many obstacles to high-quality research, including variable data quality and disparities in resources, systems and delivery of emergency care. There are also differences in infrastructure to conduct research, monitor clinical practice and/or translate findings into practice.

To address these obstacles and the relative rarity of severe outcomes in children seeking emergency care, several multicentre paediatric emergency research networks have been developed in the past two decades. These networks originally developed in high-income settings in North America (Canada, USA), Europe and Australasia (Australia and New Zealand).^1^ More recently, further networks have developed in Spain, the UK/Ireland^2 3^ and Central/South America.^4^ Together, these networks collaborate within the Paediatric Emergency Research Networks (PERN), which is currently facilitating multinational studies on topics such as asthma, sepsis and bronchiolitis.^5^

However, research conducted in high-income countries is not necessarily relevant or applicable to low- and middle-income countries (LMICs) due to differences in epidemiology, disease severity (higher burden of critical illness), resource limitations and low availability of prehospital care. For example, in status epilepticus, the median time of seizure duration prior to hospital arrival was 45 min with virtually no deaths in Australia and New Zealand,^6^ whereas in India the median time of seizure duration prior to hospital arrival was 172 min with mortality 4.6%.^7^ Delayed transport times and limited trained prehospital personnel may result in poor outcomes.

Paediatric emergency care in India is undergoing a period of rapid growth, with expansion of training, systems and protocols.^8^,^14^ In recent years, there has been increasing interest in research and growth of academic paediatric emergency medicine (PEM). A key step in the establishment of other successful PEM networks has been the development of a list of prioritised research questions.^23 15^,^18^ These prioritised questions have been useful to focus limited research resources on high-yield and clinically important problems and to drive a research agenda.^18^ By focusing on local needs, the Paediatric Resuscitation and Emergency Research Network of India (PRERNI) network aims to address the significant gaps in paediatric emergency care. This work must address resource limitations and the high burden of critical illnesses that distinguish India’s healthcare landscape from high-income countries.

A preconference paediatric emergency research workshop was held at the Society for Emergency Medicine in India conference (EMCON2023) in October 2023. A stated objective of the meeting was to develop collaborations and set priorities for a planned PEM research network for India. These initial research topics were targeted towards clinician-researchers working in emergency medicine and aimed to improve the clinical care of children attending emergency departments across India. During the workshop, a prioritisation exercise was conducted using the nominal group technique (NGT).^19^ The research network name was decided based on a survey distributed to participants following the workshop. This paper describes the methodology used and reports the research priorities identified.

## Methods

An NGT approach was used to set research priorities within the time allocated to the 1-day workshop. Key components of the NGT include (1) generating ideas; (2) recording ideas; (3) discussing and clarifying ideas and (4) voting on ideas. Each of these steps occurred throughout the workshop, interspersed with didactic talks regarding study design and research network set-up. We did not use a specific framework to set priorities. The project is reported according to the Reporting Guideline for Priority Setting for Health Research.^20^

Although Delphi studies (which use multiple survey rounds from a group of experts) are a popular method of setting priorities,^18^ the NGT approach was chosen due to the limited time frame available. Benefits of the NGT include balanced participation across members and individuals, limited interaction (which reduces the pressure to ‘conform’ with ideas from other group members), and production of a greater number of ideas than a traditional interacting group.^19^

The research and priority-setting workshop was conducted by four representatives of the Paediatric Research in Emergency Departments International Collaborative (PREDICT) Network. PREDICT, established in 2004,^18^ has conducted a number of large multicentre studies in paediatric emergency care across Australia and New Zealand.^21^,^23^ All four PREDICT representatives (FEB, EL, SRD and SC) had previously been involved in coordinating and participation in prioritisation studies for paediatric emergency research,^18 24^ and all four held executive roles within the network (chair, vice-chair, founding chair and past chair).

The workshop was advertised on the conference website and through personal contacts of prominent PEM specialists in India. Participants were selected to represent a diverse range of geographic regions, institutions and clinical roles, ensuring broad input. Workshop participants were advised that the workshop programme would include capacity-building through didactic talks and interactive sessions, as well as developing a prioritised set of research questions relevant to PEM research in India.

Stage I and II–generating and recording ideas: After a brief didactic presentation regarding the important component of research questions (clear definitions for ‘PICO’ components: Participants, Interventions, Comparators, Outcomes), workshop participants were asked to discuss / workshop ideas in small groups of 2–3, and to individually record their ideas using an online survey (Surveymonkey.com). Research questions were recorded in a series of open-ended questions, with space provided for each component of a typical ‘PICO’ question. There was no restriction on the number of questions which could be nominated, and workshop participants were allowed approximately 30 min to enter their research questions.

*S*tage III–discussing and clarifying ideas: The research questions were extracted from the electronic survey by SC and refined collaboratively. Duplicate questions were removed, and all questions were then presented to the workshop participants. Participants were asked to determine whether the questions ‘made sense’ as written, whether any modifications were required, and whether the proposed question should be split into two or more questions. Where possible, a PICO format was used for recording the proposed research topics. The process of discussion and clarification took approximately 1 hour to complete.

As this was an initial prioritisation exercise conducted during a single day, we did not conduct a systematic review to determine whether each research topic had already been answered.

Stage IV*–*voting on ideas: Each finalised research question was then presented in a second electronic survey distributed to workshop participants. Each question was accompanied by a request to rate the importance of the research question on a 9-point scale. Rankings of these questions were then used to generate the final prioritised list. We did not apply a prespecified threshold to exclude any research topics/questions.

### Role of the funding source

No specific funding was provided for this project. Individual investigators (FEB, SRD and EL) receive funding to support their research time. The funders of the study had no role in study design, data collection, data analysis, data interpretation or writing of the report.

## Results

34 workshop participants participated in the priority setting process. Of these, 33 were from India and 1 was from Indonesia. Participant details are summarised in table 1. The four PREDICT representatives provided support, facilitated the discussion and clarification of research questions and coordinated the surveys but did not contribute any research ideas or vote.

**Table 1:** Workshop participants

	n (%)*
Region/state	
India	33 (97.1)
Delhi	1 (2.9)
Karnataka	8 (23.5)
Punjab	2 (5.9)
Rajasthan	1 (2.9)
Tamil Nadu	15 (44.1)
Telangana	6 (17.6)
Indonesia	1 (2.9)
Gender	
Female	17 (50)
Male	14 (41.2)
Not specified	3 (8.8)
Role	
Professor/assistant/associate professor	18 (52.9)
Consultant	11 (32.4)
Junior doctor/student	5 (14.7)
Clinical experience	
<5 years	1 (2.9)
5−9 years	10 (29.4)
10−14 years	7 (20.6)
15−19 years	8 (23.5)
≥20 years	6 (17.6)
Not specified	2 (5.9)

**Figure 1:**
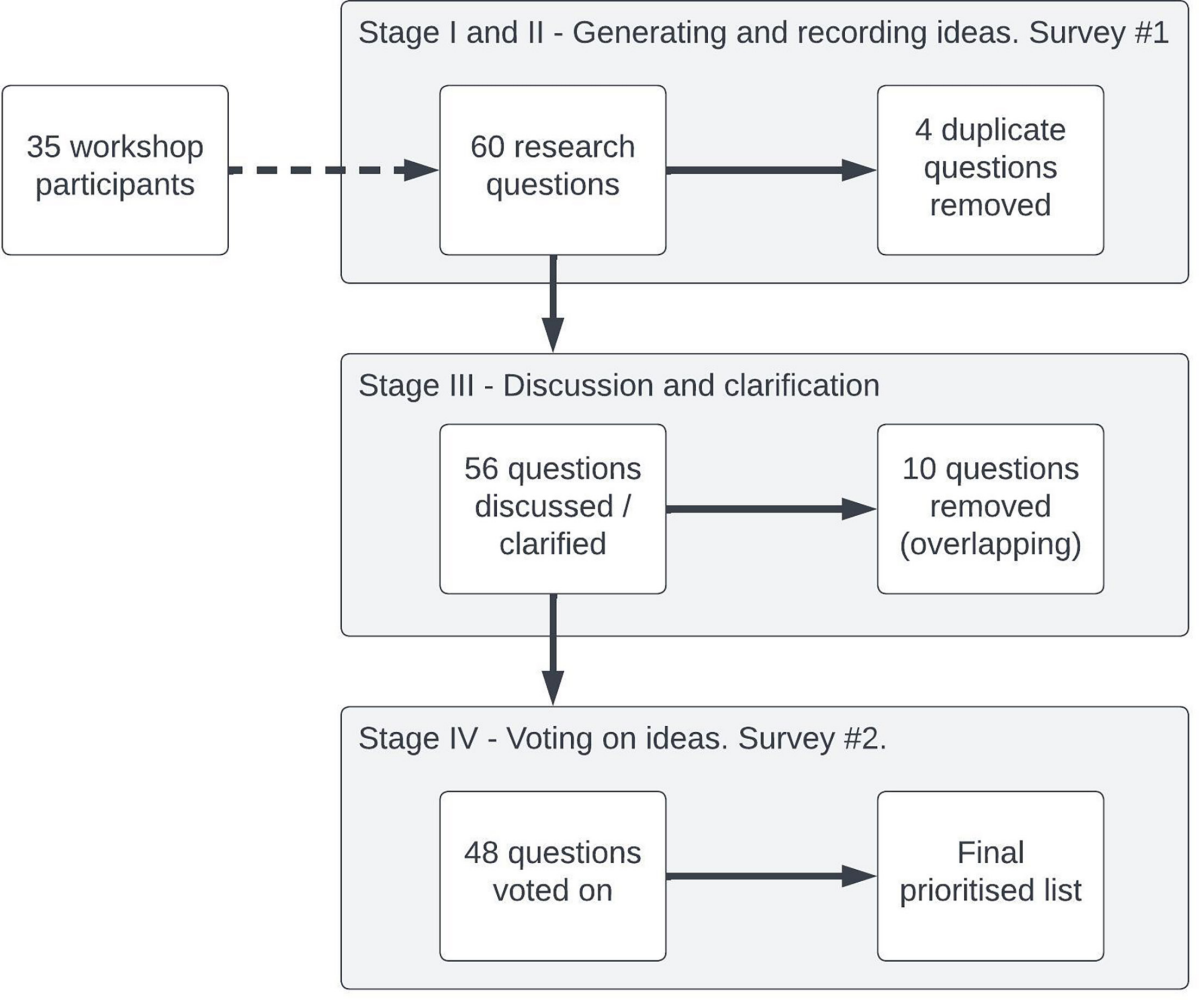
Process of developing list of research questions.

60 research questions were generated in the initial survey. Of these, four duplicate questions were removed, with 56 retained for discussion. After discussion, reframing and clarification, 10 questions were removed and 2 were added, leaving a final 48 questions for voting (figure 1). These questions were edited by the PREDICT representatives into either PICO (Population, Intervention, Comparator, Outcome) questions where there was a clear comparative trial proposed or questions suitable for observational studies. 34 workshop participants voted on the proposed research questions. Process of developing list of research questions is shown in figure 1.

The top 20 research questions are presented in table 2, while the complete list is presented in online supplemental table 1.

**Table 2:** Top 20 research priorities for paediatric emergency medicine in India

Rank	Research question	Priority score
1	In children transported to hospital, does a standardised protocol for assessment/identification and intervention prehospital, compared with no prehospital care or non-standardised care, result in improvements in mortality, hospital length of stay and overall clinical improvement?	7.58
2	What are the prehospital predictors of clinical outcome with prehospital presentation of unresponsiveness/seizures?	6.97
3	In children presenting to the ED, is the use of bedside echocardiography to assess for myocardial dysfunction superior to clinical and laboratory assessment of myocardial dysfunction in achieving clinically important outcomes?	6.93
4	In children with fluid-refractory septic shock (ie, after 40–60 mL/kg fluid boluses), is norepinephrine infusion titrated to response superior to epinephrine infusion titrated to response for achieving clinical outcomes?	6.86
5	What is the impact of a quality improvement initiative aimed at prehospital providers (which involves objective assessment of skills, addressing any deficits and reaudit) on clinical outcomes?	6.81
6	In children presenting to the ED with cardiac arrest, is intubation after optimisation (PREM intubation bundle) superior to immediate intubation for achieving clinically important outcomes?	6.8
7	In children with fluid-refractory septic shock, is early low-dose vasopressin infusion+epinephrine infusion superior to epinephrine infusion alone for achieving clinically important outcomes?	6.75
8	What are the current levels of training, skills and knowledge of prehospital providers?	6.74
9	What are the predictors of clinical outcomes of children with envenomation?	6.63
10	What are the predictors of clinical outcomes of children receiving prehospital treatment and/or transport?	6.55
11	In children presenting to the ED with unresponsiveness, is delayed intubation with period of bag-valve-mask ventilation, compared with immediate intubation, result in superior clinical outcomes?	6.52
12	What are the barriers to optimal prehospital treatment/transport?	6.5
13	What is the optimal method to risk-stratify trauma patients to determine appropriate treatment and transport?	6.42
14	How do we improve care for snake envenomation?	6.41
15	In children presenting to the ED with shock, is the use of a structured template for rapid cardiopulmonary cerebral assessment, compared with standard paediatric case record, superior for achieving a reduction in mortality?	6.4
16	How do we improve care for trauma in children?	6.27
17	What are the barriers to the optimal treatment of snake envenomation in children?	6.26
18	In children with acute respiratory distress and hypoxia, which of (a) high-flow nasal cannula, (b) Jackson-Rees circuit or (c) non-rebreather mask results in superior clinical outcomes?	6.24
19	In children presenting with respiratory failure and/or cardiac arrest, is pharmacological-assisted intubation, compared with no treatment (intubation without medications), superior for achieving successful airway management and other clinically important outcomes?	6.21
20	What are the barriers to the optimal treatment of trauma in children?	6.16

ED, emergency department; PREM, Paediatric Resuscitation and Emergency Medicine.

### Comparison with research priorities from other PEM networks

Table 3 shows the topics and rankings from the prioritisation process, compared with prior research priority setting initiatives for paediatric emergency care in other networks. This was most recently completed by Velasco and colleagues in 2023 after the development of priorities by the Spanish research network (RISEUP-SPERG).^3^

**Table 3:** Comparison between the top five PRERNI priorities and those published by other research networks (Adapted from Velasco *et al*)^3^

	PECARN Miller *et al*^15^	PERUKI Hartshorn *et al*^16^	PREDICT Deane *et al*^18^	PERC Bialy *et al*^17^	REPEM Bressan *et al*^2^	RISeuP-SPERG Velasco *et al*^3^	PRERNI
1	Respiratory illnesses/asthma	Biomarkers in fever	Asthma	Mental health presentations	Biomarkers in sepsis	Quality of care	Prehospital care
2	Prediction rules for high-stakes/low-likelihood diseases	Major trauma	Urinary tract infection	Pain and sedation	Risk stratification in sepsis	Triage	Septic shock
3	Medication error reduction	Sepsis	Major trauma	Practice tools	Practice variation in sepsis	Sedoanalgesia for procedures	Airway management during resuscitation
4	Injury prevention	Asthma	Cardiopulmonary resuscitation	Quality of care delivery	Practice variation in fever	Meningitis, encephalitis	Assessment of myocardial dysfunction
5	Urgency and acuity scaling	Clinical decision support tools	Septic arthritis/osteomyelitis	Resource utilisation	Biomarkers in fever	Clinical decision support tools	Envenomation

PECARN: PERUKI: PREDICT: PERC: REPEM: RISeuP-SPERG: PRERNI.

PECARN, Pediatric Emergency Care Applied Research Network; PERC, Pediatric Emergency Research Canada; PERUKI, Pediatric Emergency Research United Kingdom and Ireland; PREDICT, Paediatric Research in Emergency Departments International Collaborative; PRERNI, Paediatric Resuscitation and Emergency Research Network of India; REPEM, Research in European Pediatric Emergency Medicine; RISeuP, Red de Investigacion de la sociedad Espanola de Urgencias de Pediatria; SPERG, Spanish Pediatric Emergency Research Group.

The priorities identified in our study, the first for PEM from an LMIC, have some similarities with those developed in other, predominantly high-income, settings. Although sepsis has been noted as a priority by PERUKI and REPEM, the use of biomarkers to identify sepsis and/or risk-stratify children with potential sepsis was emphasised, while in the Indian setting, the management of severe sepsis/septic shock was prioritised. The PREDICT network identified cardiopulmonary resuscitation as a research priority, which has some overlap with our priority relating to airway management during resuscitation. Prehospital care, envenomation and assessment of myocardial dysfunction were not mentioned in the top priorities of other PEM research networks. However, prehospital care and ensuring competency in performance of life-saving procedures were prioritised in a modified Delphi process for general (adult and paediatric) emergency research conducted in South Africa.^25^

### Evaluation and feedback

26 workshop participants responded to the feedback survey, which sought feedback on the overall workshop, the prioritisation process and the final list of research questions. Overall, the workshop was rated highly. The prioritisation process generated mostly positive comments (eg,*“The process of collecting feedback was a novel method of immediately assessing the collective thoughts of the entire group of participants.”*). However, there were some concerns about potential biased selection of topics, as they relied on the interests of the people participating in the workshop, and did not necessarily consider epidemiology or burden of disease in India (*“A survey before the onset of the workshop on key potential areas where we could include opinions of experts who are unable to attend the workshop.”* and *“Should consider the nation-wide mortality data also.”*).

## Discussion

This study has, for the first time, identified multicentre research priorities in PEM for India. The presented list of research questions will guide research efforts over the coming years and form the basis for the development of a multicentre research network. Top-ranking areas included prehospital care, septic shock, envenomation, timing of and physiological optimisation prior to intubation, point-of-care ultrasound, the role of structured clinical assessment and trauma. The prioritisation of prehospital care reflects the urgent need to address delayed interventions, particularly in rural areas with limited prehospital care resources.

Previously published research priorities from PEM networks in high-income settings emphasise risk stratification and diagnosis of potential life-threatening conditions, perhaps reflecting the relatively low incidence of such conditions in these settings, as well as quality of and variation of care. However, most of the prioritised questions from Indian clinicians related to resuscitation of critically ill children. For example, while European and UK/Ireland^16^ networks highlighted a research focus on biomarkers for sepsis, PRERNI priorities emphasise management of septic shock. This likely reflects the much higher incidence of critical illness in the Indian setting, and differences in the role of emergency medicine, which is mostly focused on high-acuity resuscitation.

The Paediatric Resuscitation in Emergency Medicine approach is being promoted across parts of India with the intent to improve early recognition and guide resuscitation of children with critical illness.^13 26^ This approach is somewhat different to Advanced Paediatric Life Support algorithms,^27^ used predominantly in high-income countries, and may be more suitable for low-resource settings. However, despite evidence of reductions in under-5 mortality in Tamil Nadu (where the approach originated and is becoming established), there is enthusiasm to generate more definitive evidence of which components are most effective.

Envenomation, particularly snake envenomation, is a cause of significant morbidity and mortality in India. Some of this has been attributed to lack of trained health providers and/or poor utilisation of anti-venom, with current research actively aiming to build capacity to improve these clinical deficits.^28^ Trauma and sepsis, other conditions with high burden of disease, are major causes of paediatric emergency presentations and prehospital transport. An observational study of over one million paediatric prehospital care records from eleven states and one union territory in India found the most common conditions, chief complaints to be fever (22.5%), trauma (21%) and respiratory distress (14.6%), with nearly two-thirds of patients from rural areas, and more than three-quarters with socioeconomic disadvantage.^29^

Historically, India’s prehospital/emergency medical services are hampered by limited resources, variable regulation and oversight, inconsistent training and certification, and lack of access in some areas of the country.^9 29 30^ These issues likely contribute to preventable morbidity and mortality.

### Next steps for PEM research in India

Research priorities from developing nations are geographically limited to specific issues more prevalent in those regions of the world.^31^ This set of research priorities is only the beginning of multicentre research in India. Specific challenges include significant clinical workload, little protected research time, differences in resources available to private and government hospitals, the challenges of ethics and governance for multicentre research, a competitive research funding environment and difficulty accessing research infrastructure and expertise (eg, biostatistics, health economics, implementation science). In the near future, some of these challenges can be addressed through developing relationships and working with established PEM research networks outside India. However, a medium-term objective is to build capacity and develop ‘in house’ expertise in all of these areas.

Initial research projects need to account for these challenges. Critical early projects may include establishing current epidemiology and practice patterns with the use of surveys and retrospective observational studies. These will then lay the groundwork for more ambitious studies such as multicentre randomised trials.

### Limitations

The main limitation of our study is the inclusion of participants from a single workshop at an emergency medicine conference. This method was chosen to make the most of limited opportunities for interaction with the PREDICT team and the lack of an overarching body responsible for PEM training or research across India. The NGT is a robust, pragmatic method to achieve consensus and was appropriate for use to generate and prioritise an initial list of research questions.

Despite this, we acknowledge potential regional/geographic bias due to the location of the conference (South India) and the inclusion of some relatively junior clinicians with limited PEM experience. Some clinicians may also work in other parts of the hospital outside the emergency department; however, this information was not specifically collected, and if present, this is likely to reflect the diverse workforce catering to paediatric emergency cases in India.

Local priorities may not be applicable nationally. However, the highest-priority clinical areas (resuscitation, airway management, sepsis) align with priorities internationally 31 while prehospital care and envenomation reflect challenges in the LMIC setting.

The limited time for the workshop may have constrained broader participation, and the focus on urban institutions could underrepresent rural perspectives.

It is recognised that multicentre epidemiological studies generating local information on burden of disease and mortality, as well as surveys of the wider Indian PEM community, will be required to further clarify the initial priorities articulated in this paper. Future steps include expanding stakeholder engagement and piloting studies based on the prioritised research topics.

## Conclusions

As PEM research develops in India, it will be important to ensure that research activities address emergency care across the breadth of the population—from well-resourced private hospitals through to the less-resourced government hospitals where most of the population is seen. Engaging with government, consumers, clinicians and researchers across the country will be important to ensure that we are able to make a positive impact on clinical care and patient outcomes.

As the network grows and matures, and initial projects provide information regarding epidemiology and practice variation, it is likely that further research ideas will be generated and further prioritisation will be necessary. Despite this, the initial set of research priorities is all relevant to front-line clinicians caring for children across India and provide a clear initial direction to focus limited resources.

By addressing these priorities, PRERNI can drive policy changes that improve paediatric emergency outcomes across India. Collaboration with government bodies and international networks will be critical for scaling these initiatives.

### Author reflexivity statement

The research team has engaged with this statement. The research partners have co-developed the research study. This study addresses priority research questions for the LMIC partner. The first author is a LMIC partner. There are five LMIC early career researchers incorporated as authors. Data was shared with LMIC partners via email. There is no open access funding. Authors 27 to 30 did not participate in the generation and evaluation of the research questions.

## Supplementary material

10.1136/bmjph-2024-001967 online supplemental file 1

## Data Availability

Data are available on reasonable request. All data relevant to the study are included in the article or uploaded as supplementary information.
