# Supplementary material for: What are the initial research priorities for paediatric emergency medicine in India? A prioritisation study
Source: BMJ Public Health. 2026 Jun 9;4(2):e001967. doi: 10.1136/bmjph-2024-001967 (PMC13264851; doi:10.1136/bmjph-2024-001967)
Supplement: online supplemental file 1 [file bmjph-4-2-s001.docx]

**SUPPLEMENTARY TABLE: All research priorities which were considered for and voted on to determine the PEM research priorities for India.**

| **Rank** | **Research question** | **Priority score** |
| --- | --- | --- |
| 1 | In children transported to hospital, does a training EMS personnel and developing transport protocols compared to no pre-hospital care OR non-standardised care, result in improvements in mortality, hospital length of stay (LOS), and overall clinical improvement? | 7.58 |
| 2 | What are the pre-hospital predictors of clinical outcome with prehospital presentation of unresponsiveness / seizures? | 6.97 |
| 3 | In children presenting to the ED, is the use of bedside echocardiography to assess for myocardial dysfunction superior to clinical and laboratory assessment of myocardial dysfunction in achieving clinically important outcomes? | 6.93 |
| 4 | In children with fluid-refractory septic shock (i.e. after 40-60 mL/kg fluid boluses), is noradrenaline infusion titrated to response superior to epinephrine infusion titrated to response superior for achieving clinical outcomes? | 6.86 |
| 5 | What is the impact of a quality improvement initiative aimed at pre-hospital providers (which involves objective assessment of skills, addressing any deficits, and re-audit) on clinical outcomes? | 6.81 |
| 6 | In children presenting to the ED with cardiac arrest, is intubation after stabilizing patients before intubation (PREM intubation bundle) superior to immediate intubation for achieving clinically important outcomes? | 6.8 |
| 7 | In children with fluid-refractory septic shock, is early low-dose vasopressin infusion + epinephrine infusion superior to epinephrine infusion alone superior for achieving clinically important outcomes? | 6.75 |
| 8 | What are the current levels of training, skills, and knowledge of pre-hospital providers? | 6.74 |
| 9 | What are the predictors of clinical outcomes of children with envenomation? | 6.63 |
| 10 | What are the predictors of clinical outcomes of children receiving pre-hospital treatment and/or transport? | 6.55 |
| 11 | In children presenting to the ED with unresponsiveness, is delayed intubation with period of bag-valve-mask ventilation, compared to immediate intubation, result in superior clinical outcomes? | 6.52 |
| 12 | What are the barriers to optimal pre-hospital treatment / transport? | 6.5 |
| 13 | What is the optimal method to risk-stratify trauma patients to determine appropriate treatment and transport? | 6.42 |
| 14 | How do we improve care for snake envenomation? | 6.41 |
| 15 | In children presenting to the ED with shock, is the use of a structured template for rapid cardiopulmonary cerebral assessment, compared to standard paediatric case record, superior for achieving a reduction in mortality? | 6.4 |
| 16 | How do we improve care for trauma in children? | 6.27 |
| 17 | What are the barriers to the optimal treatment of snake envenomation in children? | 6.26 |
| 18 | In children with acute respiratory distress and hypoxia, which of (a) high-flow nasal cannulae, (b) Jackson-Rees circuit or (c) non-rebreather mask results in superior clinical outcomes? | 6.24 |
| 19 | In children presenting with respiratory failure and/or cardiac arrest, is pharmacological-assisted intubation, compared to no treatment (intubation without medications), superior for achieving successful airway management and other clinically important outcomes? | 6.21 |
| 20 | What are the barriers to the optimal treatment of trauma in children? | 6.16 |
| 21 | How do we improve care for children requiring pre-hospital treatment / transport? | 6.13 |
| 22 | What is the relationship between paediatric trauma scores and clinical outcomes? Are trauma scores reliably able to predict the need for intensive care unit admission? | 6 |
| 23 | In children with traumatic brain injury requiring intubation, is the use of ketamine, compared to the use of fentanyl, superior to achieve clinically important outcomes such as mortality, hospital LOS, neurodisability, and intracranial pressure? | 6 |
| 24 | In children with near-fatal asthma, is the use of subcutaneous adrenaline alongside standard care, compared to intravenous magnesium alongside standard care, superior to achieve clinically important outcomes? | 5.97 |
| 25 | In children with moderate-to-severe bronchiolitis, which of (a) normal saline nebulisation, (b) hypertonic saline nebulisation (c) epinephrine nebulisation or (d) no nebulisation are superior to achieve clinically important outcomes (such as hospital LOS, escalation of care, mortality, and intensive care unit admission)? | 5.97 |
| 26 | In children with mild diabetic ketoacidosis receiving subcutaneous insulin, is the use of standard maintenance fluids, compared to fluids administered at a rate to correct dehydration over 48 hours, superior to achieve clinically important outcomes? | 5.96 |
| 27 | Which parenteral bronchodilator provides the best clinical outcomes for children with acute severe asthma? | 5.94 |
| 28 | In children presenting to the ED with cardiac arrest, is the initial use of bag-valve mask ventilation, compared to immediate intubation, superior to achieve clinically important outcomes such as mortality? | 5.93 |
| 29 | What is the impact of clinical guidelines for pre-hospital / transport of unwell children? | 5.92 |
| 30 | In children presenting to the ED, is the use of focused triage questions and the PREM triangle, compared to standard triage assessment processes, superior to predict the likelihood of intensive care unit admission? | 5.81 |
| 31 | In children presenting to the ED, is the use of focused triage questions and the PREM triangle, compared to standard triage assessment processes, superior to achieve clinically important outcomes? | 5.77 |
| 32 | In children aged 2 months to 12 years with status epilepticus, is the use of IV levetiracetam, compared to IV sodium valproate, superior to achieve clinically important outcomes such as cessation of seizures, mortality, ICU admission, and hospital length of stay? | 5.69 |
| 33 | In children with traumatic brain injury deemed to require treatment of raised ICP, is the use of mannitol, compared to the use of hypertonic saline, superior to achieve clinically important outcomes such as mortality, hospital LOS, and neurodisability? | 5.62 |
| 34 | Is there a bedside score for respiratory distress that predicts clinical outcomes? | 5.59 |
| 35 | In children with asthma, is the use of ketamine and magnesium, compared to magnesium alone, superior to achieve clinically important outcomes? | 5.59 |
| 36 | In children requiring intubation, is intubation using a stylet, compared to intubation without a stylet, superior to achieve successful intubation and other clinically important outcomes? | 5.55 |
| 37 | In children presenting to the ED deemed to require IV fluid bolus, is the administration of 10 mL/kg, compared to administration of 20 mL/kg, superior to achieve clinically important outcomes (such as mortality, hospital length of stay, and lower rates of pulmonary oedema)? | 5.5 |
| 38 | In children with out of hospital cardiac arrest, is the use of POCUS-guided CPR, compared to standard care (CPR without POCUS assistance), superior to achieve clinically important outcomes? | 5.47 |
| 39 | In children with acute asthma exacerbations, is the use of subcutaneous adrenaline alongside standard treatment, compared to standard treatment alone, superior to achieving overall clinical improvement? | 5.4 |
| 40 | What is the accuracy of clinical, POCUS and laboratory assessment of dehydration compared to a gold standard (i.e. change in weight)? | 5.38 |
| 41 | What are the current levels of training, skills, knowledge, and prevalent practice of paediatric emergency medicine staff? | 5.38 |
| 42 | In mothers with premature rupture of membranes transferred to tertiary hospital, is the administration of ampicillin IV 2g, compared to no treatment, superior to achieving reductions in early onset sepsis in newborns within the first week of life? | 5.3 |
| 43 | In children aged 5-18 years with mild asthma / wheeze, is the use of (a) nebulised medications (without oxygen), (b) MDI route of nebulisations, or (c) nebulised medications (with oxygen), superior to achieving clinically important outcomes such as ICU admission, hospital length of stay, and overall clinical improvement within 24 hours? | 5.13 |
| 44 | In children aged 1 month to 5 years with recurrent wheezing and respiratory distress, is the use of high flow nasal cannulae, compared to usual care alone, superior in achieving clinically important outcomes such as hospital length of stay, and treatment failure requiring escalation of support to CPAP or intubation? | 5 |
| 45 | In children with near-fatal asthma, is the use of intramuscular adrenaline, compared to intravenous salbutamol infusion, superior in achieving clinical outcomes such as ICU admission, hospital length of stay, and overall clinical improvement? | 4.94 |
| 46 | In children with bronchiolitis and moderate-severe resp distress who are being treating with high-flow nasal cannulae or respiratory support, is the use of a dexmedetomidine infusion, compared to placebo, superior in achieving clinically important outcomes such as ICU admission, hospital LOS, need for respiratory support, duration of HFNC, use of non-invasive ventilation, and intubation? | 4.87 |
| 47 | How can screening for tuberculosis be optimised in the ED? | 3.19 |
| 48 | What are the barriers to screening for TB in the ED? | 2.96 |
